# Subsequent Type 2 Diabetes in Patients with Autoimmune Disease

**DOI:** 10.1038/srep13871

**Published:** 2015-09-09

**Authors:** Kari Hemminki, Xiangdong Liu, Asta Försti, Jan Sundquist, Kristina Sundquist, Jianguang Ji

**Affiliations:** 1 Division of Molecular Genetic Epidemiology, German Cancer Research Center (DKFZ), Heidelberg, Germany; 2 Center for Primary Health Care Research, Lund University, Malmö, Sweden; 3 Department of Measles, National Institute for Viral Disease Control and Prevention, Chinese Center for Disease Control and Prevention, Beijing, China; 4 Stanford Prevention Research Center, Stanford University School of Medicine, California, USA

## Abstract

Immunological data show that type 2 diabetes (T2D) manifests autoimmune features. We wanted to test the association epidemiologically by assessing subsequent diagnosis of T2D following diagnosis of autoimmune disease (AId) and subsequent AId after T2D in the same individuals. Patients were identified from three Swedish health databases. A total of 32 different AId were included. Standardized incidence ratios (SIRs) were calculated for T2D diagnosis in patients with previously diagnosed AId and compared to those without a previous AId. Among a total of 757,368 AId patients, 15,103 were diagnosed with T2D, giving an overall SIR for T2D of 1.66. T2D risks were increased after 27 AIds; the highest SIRs were noted for chorea minor (8.00), lupoid hepatitis (5.75), and Addison disease (2.63). T2D was increased after 27 of 32 AIds but we were unable to control for factors such as obesity and smoking. However, the clearly increased risks for T2D in most types of AId patients, and in reverse order increased risks for AId after T2D, do not support an overall confounding by life-style factors. Mechanistic links shared by T2D, AId and life-style factors such as obesity, perhaps through chronic inflammation, may drive autoimmune activation of T2D and many AIds.

Type 2 diabetes (T2D) is characterized by insulin resistance i.e., inability of tissues to respond to insulin, and a progressive pancreatic beta cell dysfunction in response to glucose levels. The disease is thought to be caused by environmental and inherited factors in about equal proportions. Many environmental risk factors are known and they include obesity, sedentary lifestyle, stress, nutritional factors and toxins^1^,^2^. Family history is an important risk factor which has been shown in twins and singleton siblings^1^,^3^. The prevalence of T2D varies in populations and the rate has been increasing in many global populations, including Sweden^4^,^5^.

There are solid epidemiological data indicating that families of T2D patients show an excess of type 1 diabetes (T1D) which is an autoimmune disease (AId)^6^,^7^,^8^. Additionally, latent autoimmune diabetes of the adult (LADA) or of the young (LADY) show clustering with both T2D and T1D^9^,^10^. Recent data confirm some shared genetic predispositions for both types of diabetes and the classification has become difficult particularly in obese adolescents^11^. Furthermore, T2D has been found to express autoimmune characteristics, including the presence of autoantibodies against pancreatic beta cells and self-reactive T-cells. Glucose-lowering effects of some immune modulatory therapies in T2D patients have been related to the role of inflammasome pathways in T2D, shared by AIds with an inflammatory component^11^. Accordingly, associations have been observed between psoriasis and T2D, and rheumatoid arthritis and T2D^12^,^13^. A population-level source for study of disease co-morbidity is available in Sweden through the national Hospital Discharge Register, the Outpatient Register and the regional primary health care registers^14^,^15^,^16^,^17^,^18^. In the present article we study individual risks for subsequent T2D among patients diagnosed with any of 32 AIds. With a total AId patient population of 757,368 and 15,103 subsequent T2D diagnoses this is the largest study published on these diseases with the advantage that all the results emanate from a single population of medically confirmed cases in a country of high medical standard and reasonably uniform diagnostics.

## Materials and Methods

The research database used for this study is a subset of the national datasets compiled at Center for Primary Health Care Research, Lund University, Malmo. The data were analyzed anonymously because we used the registration data from several Swedish national registers. AId and T2D patients were identified from three health care databases: national Hospital Discharge Register including all hospital discharges with dates of hospitalization and diagnoses between 1964 and 2010, national Outpatient Registry from 2001 to 2010 and Primary Health Care Registry in Stockholm (2001–2007) and Region Skane (1987–2010). These databases include all patient visits for the described periods. When a person was present in multiple registers, the first diagnosis was considered. Various versions of the International Classification of Diseases (ICD) codes were used for case identification. For T2D, until 1996 ICD code identified diabetes without specifications and therefore age 40 year at first hospitalization was used to define T2D; this cutoff is also used in the Swedish Diabetes Register (https://www.ndr.nu). Those who had diabetes diagnosis before age 40 years were excluded. The ICD-10 code E11 ‘non-insulin dependent diabetes’ was used from 1997 onwards. T1D was not included among AIds because ICD-10 code ‘insulin dependent diabetes’ also includes T2D patients treated with insulin. Using code E11 for T2D would exclude T2D patients treated with insulin.

Person-years were calculated from the date of the first medical diagnosis for AId until diagnosis of T2D, death, emigration, or closing date on December 31, 2010, whichever came first. Standardized incidence ratios (SIRs) were calculated as the ratio of observed (O) to expected number of cases^19^. We used the indirect standardization method (Equation 1):
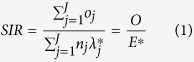


Where *O* = ∑*o*_*j*_denotes the total number of observed cases in the study group; E^*^ (expected number of cases) is calculated by applying stratum-specific standard incidence rates (λ^*^_j_) obtained from the reference group (all individuals without AId) to the stratum-specific person-years at risk (*n*_*j*_) of experience in the study group; *O*_*j*_ represents the observed number of cases that cohort subjects contribute to the *j*th stratum; and *J* represents the strata defined by the cross-classification of the following adjustment variables: age (5-year groups), sex, time period (5-year groups), socioeconomic status (6 groups), and geographic region of residence (3 groups). The 95% confidence intervals (CIs) were calculated and rounded to two decimal places for SIR, assuming a Poisson distribution. In this study, the result is statistically significant if the 95% CI does not include 1.00.

In reverse analysis the risk of AId was analyzed after T2D diagnosed at age below 60 years.

The methods in the present study were carried out in the accordance with the approved guidelines of the Regional Ethical Review Board of Lund University.

### Ethics Statement

This study was approved by the Regional Ethical Review Board of Lund University in Sweden.

The data used in the present study were obtained from several Swedish Registers and they were analyzed anonymously.

## Results

The total number of unique patients diagnosed with any of the 32 AIds was 757,368, accumulating close to 7 million person-years at risk for T2D (Table 1). The most common individual diseases were psoriasis, rheumatoid arthritis, Graves/hyperthyroidism and Hashimoto/hypothyroidism. These diseases were more common in women compared to men, similar to all AId cases. The largest female excess, 7-fold, was noted for Sjogren syndrome. The median ages for the first registered medical contacts for AId ranged from 15 years for celiac disease to 75 years for pernicious anemia.

The largest source of patient identification was the Outpatient Register which accounted for 437,000 notifications of 972,000 for all AIds (inpatients 382,000 and primary care 153,000) (data not shown). Some patients were registered in multiple sources but the number of unique AId patients was 757,368. For T2D the numbers were 132,000 of a total of 231,000 (inpatients 53,000 and primary care 46,000); the number of unique T2D patients was 168,992.

The overall risk of T2D after any AId was 1.66 (95% CI 1.63–1.68), essentially similar for men and women (Table 2). Only SIRs with statistical significance, i.e., their CIs did not overlap with 1.00, were presented in the following texts. For both sexes T2D risks were increased for 27 AIds while 5 AIs showed no increase but these had generally small numbers of cases. The largest SIRs were noted for chorea minor (8.00, 95% CI 3.81–14.77), lupoid hepatitis (5.75, 95% CI 4.46–7.29), and Addison disease (2.63, 95% CI 2.16–3.18). Clear risks of T2D were also found for common AIds (based on Table 1), such as psoriasis (2.03, 95% CI 1.96–2.10), rheumatoid arthritis (1.50, 95% CI 1.44–1.57), Graves/hyperthyroidism (1.47, 95% CI 1.41–1.54), Hashimoto/hypothyroidism (2.01, 95% CI 1.92–2.10), and ulcerative colitis (1.73, 95% CI 1.64–1.82). A significant sex-difference for T2D was observed only for chronic rheumatic heart disease (1.44, 95% CI 1.26–1.64 for men and 1.94, 95% CI 1.70–2.20 for women) and psoriasis (1.88, 95% CI 1.78–1.97 for men and 2.22, 95% CI 2.11–2.34 for women). For T2D after polymyalgia rheumatica the difference was of borderline significance (1.68, 95% CI 1.56–1.81 for men and 1.92, 95% CI 1.81–2.03 for women). For Sjogren syndrome, with an extreme female excess in case numbers, the SIRs showed no large sex differences: 1.67 (95% CI 1.29–2.12) for men and 1.50 (95% CI 1.33–1.68) for women.

Early age at T2D diagnosis was associated with an increased risk (Table 3). For all AIds the SIR was 2.43 (95% CI 2.30–2.56) when T2D was diagnosed before age 50; it was 1.78 (95% CI 1.74–1.83) when diagnosis was between 50 and 69 years and it was 1.48 (95% CI 1.44–1.51) when diagnosis was at 70+ years. At early age of onset very high SIRs were noted for lupoid hepatitis (16.85, 95% CI 8.93–28.90) and polymyositis/dermatomyositis (7.34, 95% CI 4.00–12.34).

The analysis was also carried out in reverse order. Because it was necessary to allow some follow-up time only T2D cases diagnosed before age 60 years (N = 49358) were considered (Table 4). As expected, the overall risk was highest for concurrent diagnosis (2.93, 95% CI 2.77–3.10, follow-up ≤1 year) but it was increased also for period 2–5 years (1.62, 95% CI 1.52–1.73) and for period 6+ years (1.91, 95% CI 1.77–2.06). In the latter period, SIRs were increased for 12 AIds, most for lupoid hepatitis (6.10, 95% CI 2.61–12.08), Wegener granumatolosis (5.36, 95% CI 2.84–9.19), primary biliary cirrhosis (4.97, 95% CI 2.56–8.71), and sarcoidosis (3.06, 95% CI 2.10–4.30).

## Discussion

To our knowledge this is a first nationwide attempt to assess risks for T2D after many types of AIds using unified data (and in reverse order). We used three population-level sources available in Sweden to identify patients: national Hospital Discharge Register, national Outpatient Register and a regional primary health care register. The total T2D population covered was 231,000 which is less than the year 2004 Swedish estimate at 350,000 T2D patients^20^. For year 2012 the National Swedish Diabetes Register, with some 90% coverage, reported 346,000 patients of any type of diabetes; of these were 34,000 T1D patients from the Hospital Discharge Register (www.ndr.nu). The number of T2D patients was not given but subtracting T1D patients the estimate for T2D would be around 300,000. We know that the present coverage of primary care is not complete and a proportion of persons only diagnosed in primary care would be missing. However, for the present approach it is more important that T2D diagnoses are correct rather than the coverage is complete, yet with the caveat that severe cases are most likely to be hospitalized. Because of the diagnostic codes and age limits used we assume that ‘contamination’ by T1D patients is small.

A similar discussion on diagnostic accuracy and coverage is of course relevant for AIds. Ad hoc studies from Sweden have shown 85 to 95% diagnostic accuracies for Crohn disease, ulcerative colitis, rheumatoid arthritis, celiac disease, and Wegener granulomatosis^14^,^21^,^22^,^23^. The overall diagnostic accuracy in the Swedish Hospital Discharge Register has been referred to as being 88–90% on main diagnoses^17^,^23^. With poor diagnostic accuracy any effects would be expected to regress towards null, which appeared not to be the case with the present results. Another related issue is the possibility for diagnostic and surveillance bias for antedated T2D diagnosis during treatment for AId. While some degree of this kind of bias probably usually exists for chronic diseases it is likely not to be an important contributor, for example, in view of the results in Table 3. If bias were present it should not depend on the diagnostic age of T2D. In Table 4 we see clear evidence on surveillance bias in the form of high risks of AIds in the same year when T2D was diagnosed. Another cause for co-morbidity may relate to socio-medical reasons and a tendency for patients to seek multiple medical contacts for various reasons, such as a labile disease control, referred to as ‘brittle’ diseases^24^,^25^.

The overall findings showed that T2D was increased in patients diagnosed with any of 27 AIds, and chorea minor (SIR 8.00, 95% CI 3.81–14.77) and lupoid hepatitis (5.75, 95% CI 4.46–7.29) showed the highest risks. We found no related data on lupoid (autoimmune) hepatitis but chorea minor and hemichorea have been described as a common presenting feature of metabolic disorders, including nonketotic hyperglycemia in T2D patients^26^. T2D was also increased in patients with common AIds, such as psoriasis (2.03, 95% CI 1.96–2.10), rheumatoid arthritis (1.50, 95% CI 1.44–1.57), Graves/hyperthyroidism (1.47, 95% CI 1.41–1.54), Hashimoto/hypothyroidism (2.01, 95% CI 1.92–2.10), and ulcerative colitis (1.73, 95% CI 1.64–1.82). Hazards ratios for T2D after psoriasis and rheumatoid arthritis, based on Canadian healthcare records, were increased to 1.4 and 1.5, respectively^12^. T2D risk was also increased after psoriasis but not after rheumatoid arthritis after adjustments for body-mass index (BMI), smoking and alcohol consumption in a UK study^13^. An increased odds ratio (2.81), adjusted for gender, age and weight, was reported for newly identified hypothyroidism in patients with T2D^27^. There are mechanistic links through the inflammasome pathway and many shared genes between T2D and inflammatory bowel disease but epidemiological data have been lacking^28^. In support of such sharing, several AIds were increased when these were recorded after T2D (Table 4). The highest risk (6.10, 95% CI 2.61–12.08) with follow-up of 6+ years was observed for lupoid hepatitis which strong association (5.75, 95% CI 4.46–7.29) was also noted when T2D followed lupoid hepatitis (Table 2).

The case numbers of recorded AId patients were almost twice higher for women than for men overall but for Sjogren syndrome these were 7 times higher. Yet, the overall risk of T2D was essentially similar for men and women which implied that diabetes risk was proportional to the sex-specific background incidence of AId. The only significantly different SIRs for T2D were detected for chronic rheumatic heart disease and psoriasis patients and, at borderline, for polymyalgia rheumatica patients.

Many environmental and host factors predispose to T2D and one of the weaknesses of the present study was that we were unable to control for factors such as obesity, physical inactivity, smoking, and alcohol consumption, some of which may be risk factors for certain AIds^1^,^2^. For example, smoking is a known risk factor for psoriasis and Graves/hyperthyroiditis and it is prudent to assume that smoking may contribute to the observed associations of these diseases with T2D^29^. Some AIds may involve limitations in movement and the resulting physical inactivity and weight gain may promote T2D. There are also data associating obesity and AIds^30^. Also medication for AId would be a hypothetical trigger of T2D but it would not explain the reverse associations. The previously cited study on T2D risks in psoriasis and rheumatoid arthritis patients found that adjustment for BMI, smoking, and alcohol consumption reduced the risk^13^. However, the present clearly increased risks for T2D in most types of AId patients do not support an overall role for life-style related confounding factors. Instead, we assume that there are mechanistic links shared by T2D, AId and some life-style factors such as obesity and physical inactivity. If AId and T2D would share mechanistic pathways one could assume that there might be presentation of multiple AIds in the same individual or that there would be familial clustering of several AIds. There is ample evidence on familial clustering^18^,^31^,^32^,^33^,^34^,^35^,^36^. Although mechanistic schemes are speculative and beyond this paper, we conclude by hypothesising that chronic inflammation-driven activation may be a shared initiation mechanism for T2D and many AIds^10^.

## Additional Information

**How to cite this article**: Hemminki, K. *et al.* Subsequent Type 2 Diabetes in Patients with Autoimmune Disease. *Sci. Rep.* **5**, 13871; doi: 10.1038/srep13871 (2015).

## Figures and Tables

**Table 1:** Clinical characteristics of all autoimmune diseases.

**Autoimmune disease**	**Gender (No.)**		**All (No.)**	**Median age at diagnosis**	
	**Male**	**Female**			
Addison disease	1,646	2,392	4,038	56	37,830
Amyotrophic lateral sclerosis	5,451	4,241	9,695	67	40,773
Ankylosing spondylitis	11,448	6,191	17,639	44	173,893
Autoimmune hemolytic anemia	576	831	1,407	68	13,878
Behcet disease	2,669	1,757	4,426	44	76,817
Celiac disease	15,385	26,012	41,397	15	404,585
Chorea minor	43	78	121	53	1,161
Chronic rheumatic heart disease	11,701	13,413	25,114	65	249,501
Crohn disease	21,146	22,787	43,933	41	431,430
Discoid lupus erythematosus	1,034	3,131	4,165	55	31,894
Graves/hyperthyroidism	14,875	70,764	85,639	55	955,016
Hashimoto/hypothyroidism	13,503	70,085	83,588	55	570,633
Immune thrombocytopenic purpura	3,807	4,259	8,066	43	68,014
Localized scleroderma	874	2,683	3,557	54	31,250
Lupoid hepatitis	524	1,300	1,824	54	8,055
Multiple sclerosis	8,726	17,345	26,071	48	227,157
Myasthenia gravis	1,849	2,366	4,215	62	40,877
Pernicious anemia	5,653	6,994	12,647	75	129,371
Polyarteritis nodosa	1,031	1,101	2,132	63	19,340
Polymyalgia rheumatica	17,898	34,072	51,970	73	457,669
Polymyositis/dermatomyositis	1,514	2,229	3,743	60	34,458
Primary biliary cirrhosis	684	2,942	3,626	64	20,564
Psoriasis	61,982	69,223	131,205	52	886,507
Reiter disease	597	162	759	42	7,930
Rheumatic fever	3,139	1,513	4,652	45	93,135
Rheumatoid arthritis	37,575	87,520	125,095	63	1,044,906
Sarcoidosis	11,128	10,571	21,699	48	257,076
Sjögren syndrome	1,765	12,804	14,569	60	103,979
Systemic lupus erythematosus	2,238	9,968	12,206	53	103,559
Systemic sclerosis	3,643	5,061	8,704	58	113,085
Ulcerative colitis	35,487	31,815	67,302	43	658,554
Wegener granulomatosis	5,805	11,346	17,151	72	192,329
All	279,879	477,489	757,368	56	6,995,377

Abbreviations: Pyrs, person-years.

**Table 2:** SIRs for type 2 diabetes mellitus after a specified autoimmune disease by gender.

**Autoimmune disease**	**All**			**Male**			**Female**		
	**O**	**SIR**	**95% CI**	**O**	**SIR**	**95% CI**	**O**	**SIR**	**95% CI**
Addison disease	108	**2.63**	2.16–3.18	38	**2.04**	1.44–2.80	70	**3.14**	2.44–3.96
Amyotrophic lateral sclerosis	43	0.84	0.61–1.14	29	0.83	0.56–1.20	14	0.87	0.47–1.46
Ankylosing spondylitis	314	**1.32**	1.17–1.47	223	**1.23**	1.08–1.40	91	**1.58**	1.27–1.94
Autoimmune hemolytic anemia	21	**1.77**	1.09–2.71	12	**2.04**	1.05–3.58	9	1.51	0.68–2.87
Behcet disease	81	1.24	0.98–1.54	58	1.18	0.89–1.52	23	1.41	0.90–2.13
Celiac disease	251	1.10	0.96–1.24	134	**1.22**	1.02–1.44	117	0.98	0.81–1.18
Chorea minor	10	**8.00**	3.81–14.77	4	**8.34**	2.17–21.56	6	**7.79**	2.80–17.07
Chronic rheumatic heart disease	476	**1.66**	1.51–1.82	229	**1.44**	1.26–1.64	247	**1.94**	1.70–2.20
Crohn disease	713	**1.54**	1.43–1.66	407	**1.51**	1.37–1.67	306	**1.58**	1.41–1.77
Discoid lupus erythematosus	60	1.09	0.83–1.41	22	1.15	0.72–1.74	38	1.06	0.75–1.46
Graves/hyperthyroidism	1772	**1.47**	1.41–1.54	410	**1.49**	1.35–1.64	1362	**1.47**	1.39–1.55
Hashimoto/hypothyroidism	2013	**2.01**	1.92–2.10	430	**2.09**	1.89–2.29	1583	**1.99**	1.89–2.09
Immune thrombocytopenic purpura	182	**2.46**	2.12–2.85	97	**2.42**	1.96–2.96	85	**2.51**	2.00–3.10
Localized scleroderma	75	**1.72**	1.35–2.15	22	**1.90**	1.19–2.88	53	**1.65**	1.24–2.16
Lupoid hepatitis	68	**5.75**	4.46–7.29	23	**5.41**	3.42–8.13	45	**5.94**	4.33–7.95
Multiple sclerosis	303	**1.22**	1.09–1.37	120	1.12	0.92–1.33	183	**1.30**	1.12–1.51
Myasthenia gravis	92	**1.68**	1.36–2.06	53	**1.79**	1.34–2.34	39	**1.56**	1.11–2.13
Pernicious anemia	140	**1.21**	1.01–1.42	86	**1.39**	1.11–1.72	54	0.99	0.75–1.30
Polyarteritis nodosa	52	**2.01**	1.50–2.64	30	**2.21**	1.49–3.16	22	**1.79**	1.12–2.71
Polymyalgia rheumatica	1900	**1.82**	1.74–1.91	724	**1.68**	1.56–1.81	1176	**1.92**	1.81–2.03
Polymyositis/dermatomyositis	94	**2.10**	1.70–2.58	51	**2.32**	1.73–3.05	43	**1.90**	1.37–2.56
Primary biliary cirrhosis	96	**2.18**	1.77–2.66	26	**2.63**	1.72–3.86	70	**2.05**	1.60–2.59
Psoriasis	3028	**2.03**	1.96–2.10	1579	**1.88**	1.78–1.97	1449	**2.22**	2.11–2.34
Reiter disease	14	1.20	0.65–2.02	12	1.17	0.60–2.06	2	1.40	0.13–5.14
Rheumatic fever	118	**1.32**	1.09–1.58	84	1.20	0.96–1.49	34	**1.77**	1.22–2.47
Rheumatoid arthritis	2070	**1.50**	1.44–1.57	788	**1.52**	1.42–1.63	1282	**1.49**	1.41–1.57
Sarcoidosis	646	**2.11**	1.95–2.28	346	**2.02**	1.81–2.24	300	**2.24**	1.99–2.51
Sjögren syndrome	353	**1.52**	1.37–1.69	65	**1.67**	1.29–2.12	288	**1.50**	1.33–1.68
Systemic lupus erythematosus	201	**1.83**	1.59–2.10	44	**1.50**	1.09–2.02	157	**1.95**	1.66–2.28
Systemic sclerosis	129	**1.34**	1.12–1.60	71	**1.44**	1.13–1.82	58	1.24	0.94–1.61
Ulcerative colitis	1359	**1.73**	1.64–1.82	875	**1.77**	1.66–1.89	484	**1.65**	1.51–1.81
Wegener granulomatosis	215	**1.45**	1.26–1.66	80	**1.44**	1.14–1.79	135	**1.46**	1.22–1.73
All	15103	**1.66**	1.63–1.68	6519	**1.62**	1.58–1.66	8584	**1.68**	1.65–1.72

Bold type indicates that the 95% CI does not include 1.00.

Abbreviations: O, observed; SIR, standardized incidence ratio; CI, confidence interval.

**Table 3:** SIRs for T2D after a specified autoimmune disease by age at T2D diagnosis.

**Autoimmune disease**	**<50**			**50–69**			**≥70**		
	**O**	**SIR**	**95% CI**	**O**	**SIR**	**95% CI**	**O**	**SIR**	**95% CI**
Addison disease	7	2.46	0.97–5.10	51	**3.21**	2.39–4.23	50	**2.24**	1.67–2.96
Amyotrophic lateral sclerosis	7	**4.67**	1.85–9.67	10	0.53	0.25–0.98	26	0.85	0.55–1.24
Ankylosing spondylitis	48	**2.09**	1.54–2.77	186	**1.28**	1.10–1.48	80	1.14	0.90–1.42
Autoimmune hemolytic anemia	1	1.52	0.00–8.73	10	**2.67**	1.27–4.93	10	1.34	0.64–2.48
Behcet disease	8	1.45	0.62–2.88	47	1.32	0.97–1.76	26	1.06	0.69–1.56
Celiac disease	50	**1.81**	1.34–2.38	129	**1.22**	1.02–1.45	72	**0.75**	0.59–0.95
Chorea minor	1	7.01	0.00–40.18	5	**10.19**	3.22–23.98	4	**6.48**	1.69–16.77
Chronic rheumatic heart disease	14	**2.20**	1.20–3.70	159	**2.08**	1.77–2.43	303	**1.49**	1.32–1.66
Crohn disease	100	**1.94**	1.58–2.36	392	**1.63**	1.47–1.80	221	**1.30**	1.14–1.49
Discoid lupus erythematosus	4	1.28	0.33–3.31	33	1.25	0.86–1.76	23	0.90	0.57–1.36
Graves/hyperthyroidism	132	**1.89**	1.58–2.24	714	**1.55**	1.43–1.66	926	**1.38**	1.29–1.47
Hashimoto/hypothyroidism	230	**3.89**	3.40–4.43	842	**2.19**	2.05–2.35	941	**1.68**	1.58–1.79
Immune thrombocytopenic purpura	12	**2.03**	1.04–3.55	77	**3.04**	2.40–3.80	93	**2.18**	1.76–2.67
Localized scleroderma	6	2.29	0.83–5.03	37	**2.34**	1.65–3.23	32	1.26	0.86–1.78
Lupoid hepatitis	13	**16.85**	8.93–28.90	37	**6.55**	4.61–9.03	18	**3.33**	1.97–5.27
Multiple sclerosis	47	**2.43**	1.79–3.23	152	1.08	0.91–1.26	104	1.19	0.97–1.44
Myasthenia gravis	9	**3.69**	1.67–7.03	32	**1.70**	1.16–2.40	51	**1.53**	1.14–2.01
Pernicious anemia	3	1.97	0.37–5.83	32	**1.66**	1.13–2.35	105	1.10	0.90–1.33
Polyarteritis nodosa	2	2.16	0.20–7.94	23	**2.65**	1.68–3.98	27	**1.66**	1.09–2.42
Polymyalgia rheumatica	19	**2.52**	1.52–3.95	413	**1.90**	1.72–2.09	1468	**1.79**	1.70–1.89
Polymyositis/dermatomyositis	14	**7.34**	4.00–12.34	39	**2.21**	1.57–3.02	41	**1.63**	1.17–2.22
Primary biliary cirrhosis	3	4.38	0.83–12.96	44	**2.40**	1.74–3.22	49	**1.96**	1.45–2.59
Psoriasis	343	**2.93**	2.63–3.26	1638	**2.12**	2.02–2.22	1047	**1.74**	1.63–1.84
Reiter disease	3	1.82	0.34–5.38	9	1.22	0.55–2.33	2	0.75	0.07–2.77
Rheumatic fever	7	1.17	0.46–2.43	68	**1.49**	1.16–1.89	43	1.14	0.83–1.54
Rheumatoid arthritis	88	**2.43**	1.95–2.99	761	**1.69**	1.57–1.82	1221	**1.37**	1.29–1.45
Sarcoidosis	77	**2.91**	2.30–3.64	331	**2.19**	1.96–2.44	238	**1.86**	1.63–2.12
Sjögren syndrome	22	**2.88**	1.80–4.36	175	**1.77**	1.51–2.05	156	**1.25**	1.06–1.46
Systemic lupus erythematosus	24	**3.44**	2.20–5.12	96	**1.96**	1.59–2.40	81	**1.50**	1.19–1.87
Systemic sclerosis	15	**2.26**	1.26–3.74	50	1.24	0.92–1.64	64	**1.31**	1.01–1.67
Ulcerative colitis	157	**1.94**	1.65–2.27	735	**1.90**	1.76–2.04	467	**1.46**	1.33–1.60
Wegener granulomatosis	1	0.81	0.00–4.62	48	**2.52**	1.86–3.34	166	**1.30**	1.11–1.51
All	1309	**2.43**	2.30–2.56	6524	**1.78**	1.74–1.83	7270	**1.48**	1.44–1.51

Bold type indicates that the 95% CI does not include 1.00.

Abbreviations: O, observed; SIR, standardized incidence ratio; CI, confidence interval.

**Table 4:**
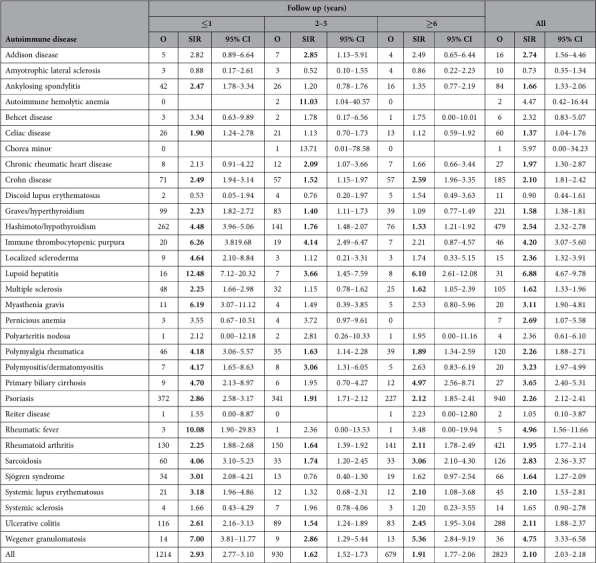
SIRs for autoimmune diseases after type 2 diabetes mellitus by follow-up time (age at T2DM diagnosis <60 years).

Bold type indicates that the 95% CI does not include 1.00. Abbreviations: O, observed; SIR, standardized incidence ratio; CI, confidence interval.
